# Mapping of the complement C1q binding site on *Trichinella spiralis* paramyosin

**DOI:** 10.1186/s13071-018-3258-x

**Published:** 2018-12-27

**Authors:** Zixia Wang, Chunyue Hao, Jingjing Huang, Qinghui Zhuang, Bin Zhan, Xinping Zhu

**Affiliations:** 1 Department of Medical Microbiology and Parasitology, School of Basic Medical Sciences, Capital Medical University, Beijing, China; 2 Department of Pediatrics, National School of Tropical Medicine, Baylor College of Medicine, Houston, Texas USA

**Keywords:** *Trichinella spiralis*, Paramyosin, Immune evasion, Complement C1q

## Abstract

**Background:**

*Trichinella spiralis* is a tissue-dwelling parasite has developed the ability to evade the host immune attack to establish parasitism in a host. One of the strategies evolved by the nematode is to produce proteins that immunomodulate the host immune system. *Ts*Pmy is a paramyosin secreted by *T. spiralis* on the surface of larvae and adult worms that can interact with complement components C1q and C8/C9 to compromise their activation and functions. To better understand the mechanism of *Ts*Pmy involved in the C1q inactivation and immune evasion, the C1q-binding site on *Ts*Pmy was investigated.

**Methods:**

The *Ts*Pmy C1q-binding site was investigated by sequential narrow-down fragment expression in bacteria and peptide binding screening. C1q binding activity was identified by Far-Western blotting and ELISA assays.

**Results:**

After several runs of sequential fragment expression, the C1q binding site was narrowed down to fragments of N-terminal *Ts*Pmy226-280aa and *Ts*Pmy231-315aa, suggesting the final C1q binding site is probably located to *Ts*Pmy231-280aa. A total of nine peptides covering different amino acid sequences within *Ts*Pmy231-280aa were synthesized. The binding assay to C1q determined that only P2 peptide covering *Ts*Pmy241-280aa binds to C1q, indicating that the C1q binding domain may need both the linearized sequence and conformational structure required for binding to C1q. The binding of peptide P2 to C1q significantly inhibited both C1q-initiated complement classical activation and C1q-induced macrophage chemotaxis.

**Conclusions:**

This study identifies the C1q binding site within *Ts*Pmy which provides helpful information for developing a vaccine against trichinellosis by targeting the C1q-binding activity of *Ts*Pmy.

## Background

*Trichinella spiralis* is a parasitic nematode that has a broad host range, including humans and more than 150 mammalian species [1]. Trichinellosis is a worldwide foodborne zoonotic disease caused by the ingestion of undercooked or raw meat infected with infective larvae of *T. spiralis* [2]. This tissue-dwelling parasite has developed the ability to evade the host immune attack in order to become established in the host [3–5]. The mechanism by which *Trichinella* evades the host immune response and survives in a hostile environment is not well understood [6, 7].

The complement has been identified as a first line immune defense system against invading pathogens. C1q is the first subcomponent of the C1 complex of classical complement pathway activation and plays a vital role in linking innate and acquired immunity. More evidence has suggested that C1q is an important target for immune evasion of some pathogens [8–15].

Paramyosin is a fibrous protein in invertebrates which is not only a structural component of muscle, but also an important immunomodulatory protein expressed on the surface of parasitic helminths [5, 16, 17]. *Ts*Pmy, the paramyosin expressed by the larval and adult *T. spiralis* [5], plays an important role in the process of the nematode’s immune evasion from host complement attack by binding to complement component C1q [4] and C8/C9 [5, 18]. It has therefore become the leading candidate for a vaccine against *T. spiralis* infection, particularly since mice immunized with different forms of *Ts*Pmy (recombinant protein, DNA, multiepitope) have shown to develop significant protection against a challenge of *T. spiralis* infective larvae [19–25]. The binding domain to C9 has been pinned down to the 14 amino acid residues within the *Ts*Pmy C-terminal region (^866^Val-^879^Met) [18]. Further studies have revealed that *Ts*Pmy’s binding to C1q inhibited not only the classical complement activation, but also C1q-induced macrophage migration and functions [4]. However, the C1q binding site on *Ts*Pmy has not been determined. Identification of the C1q binding site on *Ts*Pmy would further facilitate our understanding of the complement binding and evasion activity of *Ts*Pmy. This information will assist the design of drugs and vaccines based on its complement regulatory functions.

To ascertain the C1q binding site of *Ts*Pmy, sequential fragment expressions were performed in this study and their binding activities to C1q were investigated. As a result, the C1q binding site was narrowed down to the region between^241^Val and ^280^Ile at the N-terminus of *Ts*Pmy. The synthesized peptide based on this region was able to bind to C1q and subsequently inhibit the activation of the C1q-initiated classical pathway and C1q-induced macrophage migration. Our results provide important information to further understand the interaction between *Ts*Pmy and C1q and complement evasion mechanisms of *T. spiralis*, therefore facilitating the design of novel drugs and vaccines to control trichinellosis.

## Methods

### Sera

Normal human serum (NHS) was selected from healthy human volunteers. Human C1q-deficient serum (C1q D) was purchased from Merck (Merck Darmstadt, Germany).

### Expression of *Ts*Pmy and its fragments

To identify and locate the complement C1q binding site on *Ts*Pmy, the full-length *T. spiralis* paramyosin (*Ts*Pmy1-885aa, NCBI accession numbers for nucleotides sequence: EF429310.1 and protein sequence: ABO09862.1) was expressed as recombinant protein using a baculovirus insect expression system (Invitrogen, Carlsbad, CA, USA), and the subsequent fragments of *Ts*Pmy, with 30 amino acids overlapped (*Ts*Pmy1-315aa, *Ts*Pmy286-600aa, *Ts*Pmy571-885aa), were sub-cloned and expressed in an *E. coli* expression system (Novagen, Merck, Darmstadt, Germany) as described in [4]. Further fragmenting expression on the C1q-binding fragments of *Ts*Pmy1-315aa (*Ts*Pmy1-125aa, 96-220aa, 191-315aa) and *Ts*Pmy191-315aa (*Ts*Pmy191-245aa, 226-280aa, 261-315aa) was performed in *E. coli*. All recombinant proteins were expressed with 6-histidine tag and purified by nickel affinity chromatograph (GE Healthcare, Boston, MA, USA).

### Peptide design and synthesis

To finally determine the amino acid sequence of the C1q binding site on *Ts*Pmy, the peptides with different amino acid sequences were synthesized by solid-phase peptide synthesis (China Peptides, Shanghai, China). The acquired 9 peptides were designed (P1-P9, *Ts*Pmy231-280aa, *Ts*Pmy241-280aa, *Ts*Pmy251-280aa, *Ts*Pmy261-280aa, *Ts*Pmy231-275aa, *Ts*Pmy231-276aa, *Ts*Pmy231-277aa, *Ts*Pmy231-278aa, *Ts*Pmy231-279aa), purified up to 95% by preparative RP-HPLC and verified by mass spectrometry.

### C1q binding assay of *Ts*Pmy and its fragments

#### Far-Western blotting

To detect whether the expressed recombinant *Ts*Pmy and its fragment proteins bind to human C1q, Far-Western blotting was used based on the method as previously described [26]. Briefly, 1 μg of each protein was subject to 12 or 15% SDS-PAGE under reducing conditions, and then transferred onto a nitrocellulose membrane (GE Healthcare). After being blocked with 5% (w/v) dry milk in PBS, the membrane was incubated with human C1q (5 μg/ml in PBS, pH 7.4, 0.5 mM Ca^2+^, 0.5 mM Mg^2+^, 1% dry milk) at 37 °C for 2 h, then probed with anti-C1q antibody (Abnova, Taipei City, Taiwan, China) at a 1:10,000 dilution in the same dilution buffer for 1 h at room temperature. IRDye 800CW-labled goat anti-mouse IgG (LI-COR, Lincoln, NE, USA) at 1:10,000 was used as the secondary antibody. Bovine serum albumin (BSA) (Thermo Fisher, Life Technologies, Carlsbad, CA, USA) and *Ts*87, a *T. spiralis* expressed protein [27], were used as negative and non-relevant controls. The membranes were visualized and imaged with an Odyssey CLx Infrared Imaging System (LI-COR).

#### ELISA

An ELISA-based assay was also used to determine the C1q binding activity of *Ts*Pmy and its fragments according to the method as previously described [28]. Briefly, a 96-well ELISA plate was coated with human C1q at 5.0 ug/ml in carbonate buffer (100 mM Na_2_CO_3_/NaHCO_3_, pH 9.6), blocked with 5.0% (w/v) BSA in PBS at 37 °C for 2 h, then incubated with different concentrations of recombinant *Ts*Pmy fragments at 25 °C for 1 h. Anti-His mAb at 1:1000 in PBS/1% BSA was used to probe recombinant proteins, and HRP-conjugated goat anti-mouse IgG (BD Biosciences, Franklin Lakes, NJ, USA; 1:5000 in PBS/1% BSA) was used as secondary antibody. For the peptide binding assay, synthesized peptide P2 was labeled with biotin and the C1q coated plate was incubated with different concentrations of biotinylated peptide P2 (B-P2). HRP-conjugated streptavidin (Abcam, Cambridge, UK; 1:10,000 in PBS/1% BSA) served as the detection antibody. After adding the substrate o-phenylendiamine dihydrochloride (OPD, Sigma-Aldrich, St. Louis, MO, USA), absorbance was measured at 450 nm with a plate reader (Thermo Fisher, Life Technologies). BSA coated on the same plate was used as a negative control.

#### Dot blot

Dot blot was used to detect whether the synthesized peptides bind to human C1q. The peptides (5 μg in total volume of 2.5 ul) were spotted onto a nitrocellulose membrane. After blocking with 5% (w/v) dry milk in PBS, the membrane was incubated with human C1q (5 μg/ml) at 37 °C for 2 h, washed with PBS, then probed with anti-C1q antibody (1:10,000) at room temperature for 1 h. The same amount of C1q and BSA were used as controls.

### Detection of peptide P2-mediated inhibition of complement C4 deposition

To evaluate whether the binding of peptide P2 to C1q inhibits the C1q-initiated complement activation, the intermediate complement C4 deposition following complement activation was analyzed [29]. To initiate the classical activation, purified human IgM (Calbiochem, Merck, Darmstadt, Germany) was coated on plates (0.2 μg/well). The plates were blocked with 3% (w/v) BSA, then incubated at 37 °C for 1 h with NHS which was pre-incubated with various doses of peptide P2 (0, 2, or 6 μg) in 1× Veronal buffer (VB; Lonza, Basel, Switzerland) containing 0.05% (v/v) Tween-20 and 0.1% (w/v) gelatin. Subsequently, goat anti-human C4 mAb (1:10,000, Abcam) was added and incubated for 1 h. HRP-conjugated rabbit anti-goat IgG (1:10,000; BD Biosciences) was used as the secondary antibody. OPD was used as substrate and absorbance at 450 nm was measured.

### Hemolytic assay

To determine whether the binding of P2 peptide to C1q inhibits the classical complement activation-mediated hemolysis in the same way as the full-length *Ts*Pmy, 100 μl of fresh sheep red blood cells (SRBC 5 × 10^8^ cells/ml) were sensitized with rabbit anti-SRBC antibody (Sigma-Aldrich) at a dilution 1:200 in HBSS^++^ buffer (Hank’s Balanced Salt Solution containing 0.6 mM MgSO_4_·7H_2_O, 0.5 mM MgCl_2_·6H_2_O, 1.3 mM CaCl_2_) at 37 °C for 30 min; 2 μg C1q was pre-incubated with various amounts of peptide P2 (0, 0.5 and 1.0 μg) at 37 °C for 90 min before the addition of 100 μl of 1:50 diluted human C1q-deficient serum (C1q D). Subsequently, sensitized SRBCs were mixed with the treated C1q D at 37 °C for 30 min. Cold HBSS^++^ containing 10 mM EDTA was added to stop the reaction and the solution was centrifuged at 1000× *g* for 10 min. The absorbance of the supernatants was measured at 412 nm to calculate the reduced hemolysis compared to the total lysis in water.

### Transwell migration assay

To evaluate the effect of the C1q-binding peptide P2 on the C1q-induced migration of THP-1-derived macrophages, a cell migration assay was performed using a 24-well-transwell permeable support system with 8 μm pores (Corning, New York, NY, USA). Briefly, M2 macrophages were induced from human leukemia monocytic THP-1 cells (an acute monocytic leukemia cell line, Infrastructure of Cell Line Resource, http://cellresource.cn/contact.aspx, China) by adding phorbol-12-myristate-13-acetate (PMA) (100 ng/ml, Sigma-Aldrich) for 48 h and then human IL-4 (Prepro Tech, Rocky Hill, NJ, USA) for 24 h as previously described [30]. The THP-1-derived M2 macrophages (2 × 10^5^) were added to the upper chamber. RPMI 1640 containing human C1q (10 nM) mixed with different amounts of peptide P2 (0, 2, 4 and 6 μg) was placed in lower chamber. After incubation at 5% CO_2_, 37 °C for 24 h, the cells that migrated to the bottom surface of the membrane were stained with Giemsa and counted in 10 randomly chosen fields under a microscope as described previously [4]. *Ts*Pmy (6 μg) was added as a positive control and BSA (6 μg) as a negative control. LPS (100 ng/ml) was used as positive control to induce the migration of macrophages.

### Statistical analysis

The results are presented as the mean ± standard error of the mean (SEM) or the mean ± standard deviation (SD). The data were statistically analyzed using SPSS v.17.0 software. One-way ANOVA with LSD-*t* multiple comparison test analysis was utilized to analyze the differences between groups. *P* < 0.05 was considered statistically significant.

## Results

### Binding of *Ts*Pmy to human C1q

**Fig. 1:**
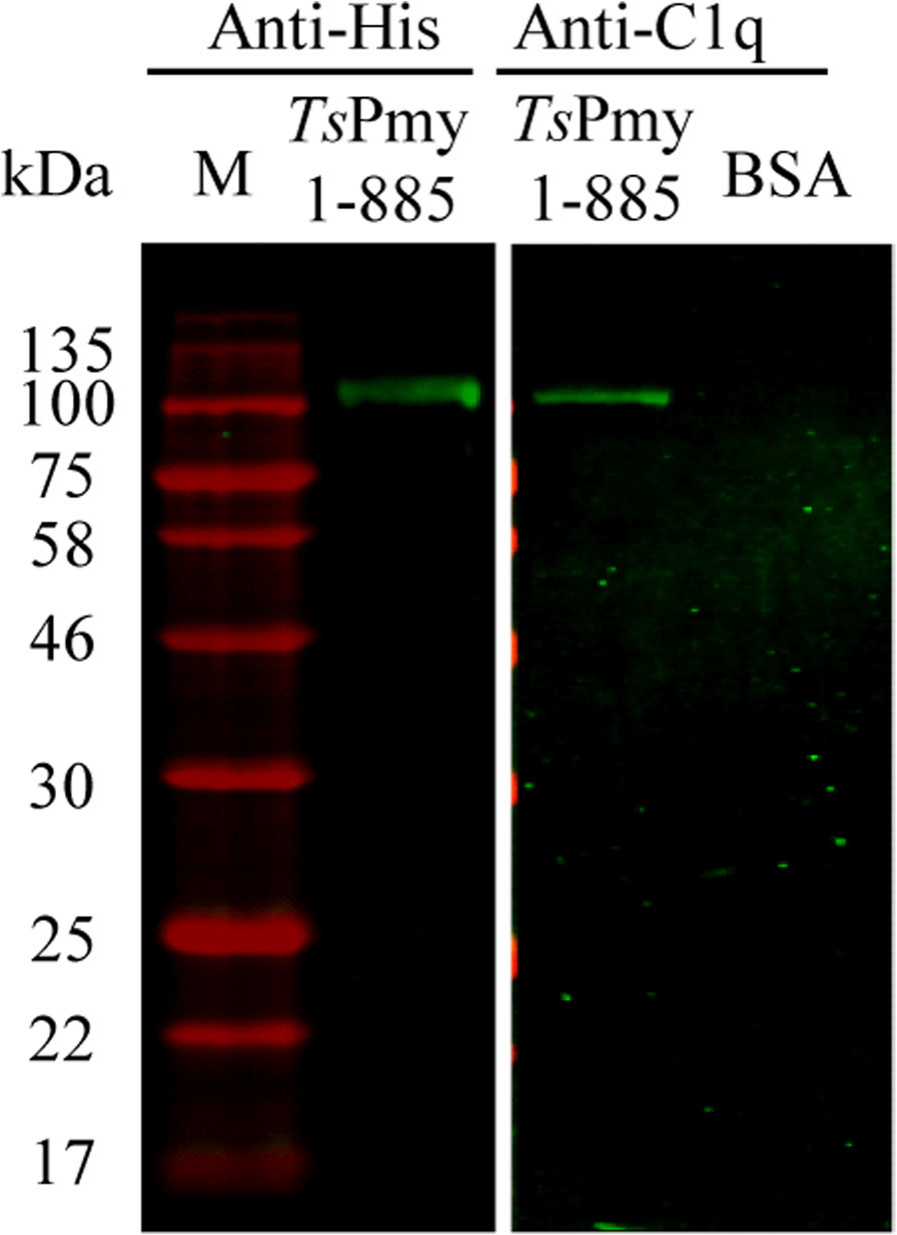
Far-Western blotting showing binding of full-length *Ts*Pmy (1-885aa) to human C1q. The membrane transferred with *Ts*Pmy1-885aa (1 μg) was incubated with human C1q (5 μg/ml) and then probed with anti-C1q antibody (1:10,000). BSA served as non-relevant control. The same amount of *Ts*Pmy1-885aa on the membrane was detected by anti-His Ab as control (~110 kDa). Results were repeated three times. Lane M: molecular weight marker

The binding of full-length *Ts*Pmy1-885aa to human complement C1q was confirmed by Far-Western blotting analysis. The results demonstrated that C1q was able to bind onto full-length *Ts*Pmy on the membrane (Fig. 1). As a control, C1q could not bind to the same amount BSA at the same condition.

### Mapping of C1q binding fragment of *Ts*Pmy

**Fig. 2:**
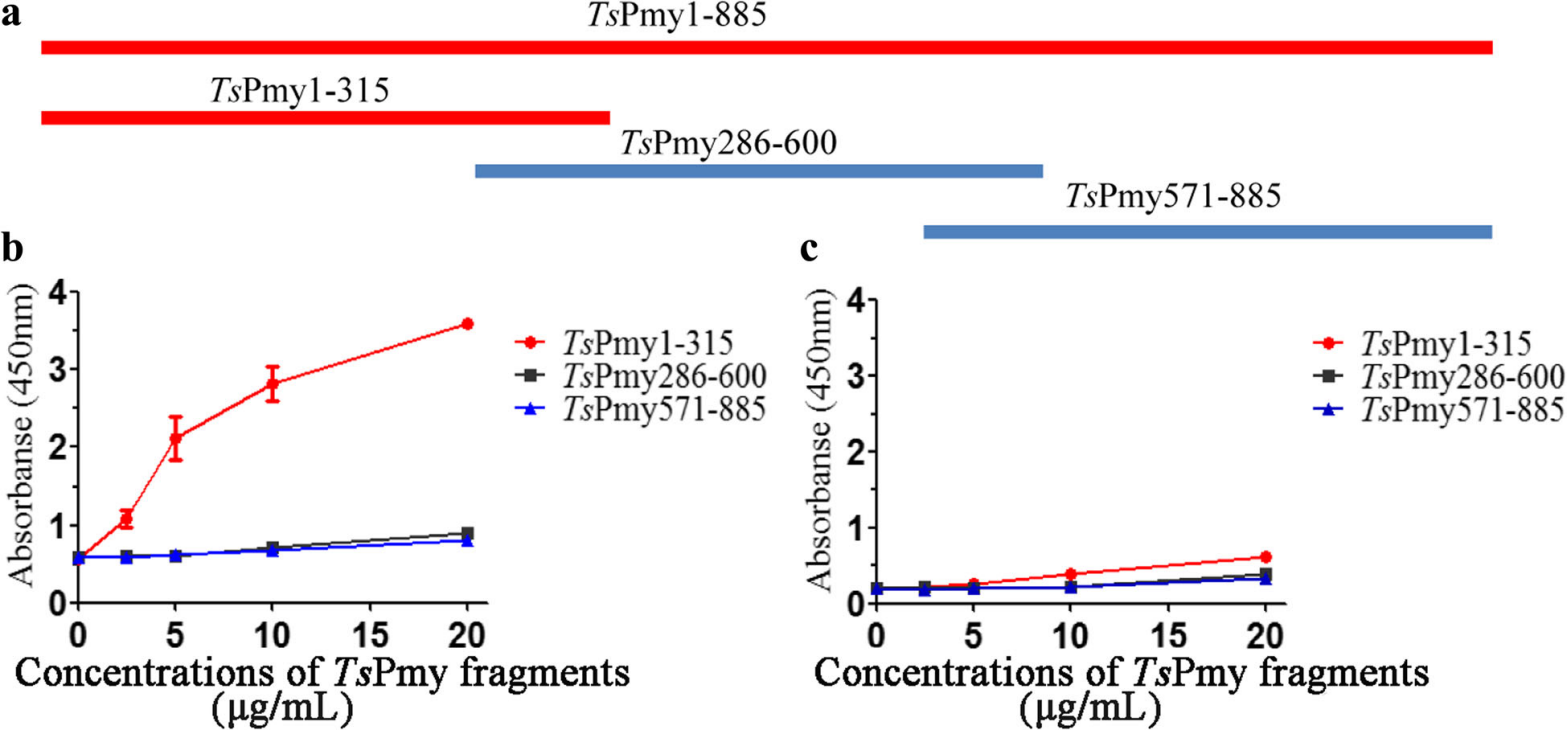
Determination of the *Ts*Pmy fragment that binds to C1q by ELISA. ELISA plate was coated with C1q (**b**) or BSA (**c**) (5 μg/ml), then incubated with different concentrations (0, 2.5, 5, 10 and 20 μg/ml) of each recombinant fragment of *Ts*Pmy (1-315aa, 286-600aa and 571-885aa) (**a**), then detected with anti-His mAb (1:1,000) (**b**, **c**). Results were repeated three times. Data are shown as mean ± SEM

**Fig. 3:**
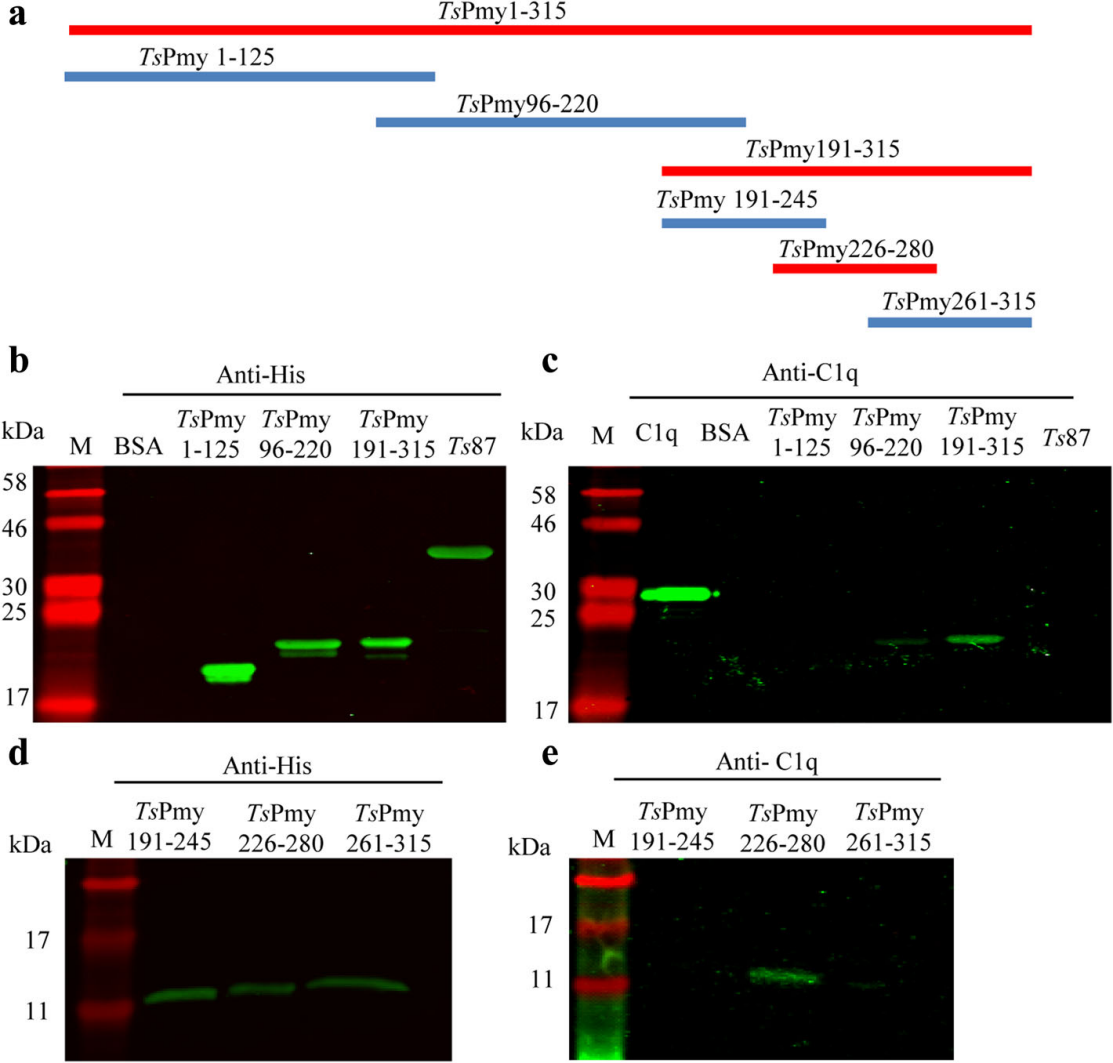
Determination of the C1q binding fragment of *Ts*Pmy within *Ts*Pmy1-315aa. **a** The diagram of fragment design within *Ts*Pmy1-315aa. **b**, **c** Far-Western blotting showing fragment *Ts*Pmy191-315aa binds to C1q. **d**, **e** Further narrow-down expression of fragments within *Ts*Pmy191-315aa showing that fragment *Ts*Pmy226-280aa confers the strong binding ability to human C1q. The fragments were incubated with human C1q (5 μg/ml) and then probed with anti-C1q antibody (1:10,000) or directly probed with anti-His Ab as control for the size of recombinant fragments with His-tag. BSA and *Ts*87 (with His-tag) served as negative control. Results were repeated three times. Lane M: molecular weight marker

To identify the C1q binding domain(s) within *Ts*Pmy, three different fragments covering the whole molecule with 30 amino acids overlapped were expressed as recombinant proteins (Fig. 2a). The binding of recombinant fragments to human complement C1q was determined by ELISA with C1q coated on a plate. Only fragment *Ts*Pmy1-315aa bound to C1q in a dose-dependent manner, detected by anti-His Ab; the rest of the fragments did not bind (Fig. 2b). All fragments did not bind to BSA coated on a plate with the same conditions (Fig. 2c). Since *Ts*Pmy 1-315aa is a fragment that binds to C1q, further fragment expressions were performed within amino acid 1-315aa (Fig. 3a). The C1q binding site was narrowed down to *Ts*Pmy191-315aa detected by Far-Western blotting with fragments transferred on a membrane which was incubated with C1q and probed with anti-C1q Ab (Fig. 3b, c). A further narrow-down fragment assay identified the final C1q-binding site was located at *Ts*Pmy226-280aa (Fig. 3d, e). ELISA with C1q coated on a plate also concluded similar results (data not shown). Another similar fragment expression approach showed that the fragment *Ts*Pmy231-315aa bound to C1q at a similar level to fragment *Ts*Pmy226-280aa (data not shown), indicating that the C1q binding site should fall into the fragment between 231-280aa. The *Ts*Pmy fragments binding to C1q was *Ts*Pmy-specific since the same amount of BSA or *Ts*87 did not show any binding activity.

### Determination of the C1q binding peptide

**Fig. 4:**
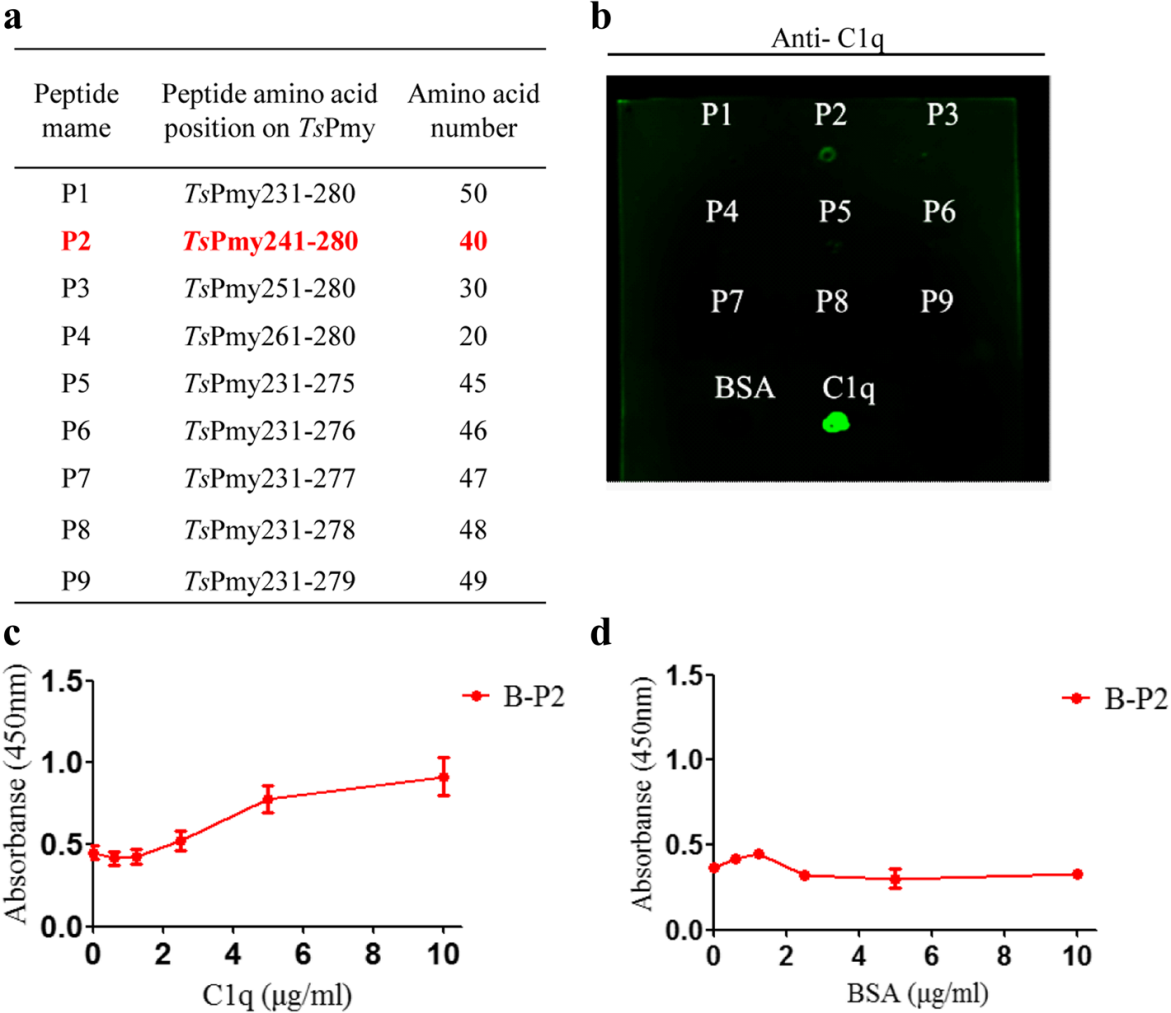
Binding ability to human C1q of synthesized peptides derived from different size of *Ts*Pmy231-280aa. **a** The amino acid sequence initiating and ending number of synthesized peptides. **b** Peptide P1 to P9 (5 μg) were spotted onto a nitrocellulose membrane and incubated with human C1q, then probed with anti-C1q antibody. The same amount of BSA and C1q were used as controls. The binding of P2 to human C1q was further confirmed by ELISA with plates coated with different concentrations of C1q (**c**) or BSA (**d**) (0, 0.625, 1.25, 2.5, 5 and 10 μg/ml), then incubated with same amount of peptide B-P2 (10 μg/ml) and detected by HRP-conjugated streptavidin (1:10,000). Results were repeated three times, ELISA data are shown as mean ± SEM

The fragment expression assay determined that the C1q binding domain should be within *Ts*Pmy231-280aa. To further pinpoint the C1q binding site within this fragment, 9 peptides with different amino acids (20–50) covering *Ts*Pmy231-280aa (P1-P9) were synthesized for evaluating their binding activity to human C1q (Fig. 4a). After being spotted onto a nitrocellulose membrane, the peptides (P1-P9) were probed with human C1q and detected with anti-C1q antibody. The dot-blot analysis demonstrated that only peptide P2 (*Ts*Pmy241-280aa) was able to bind C1q (Fig. 4b), narrowing down the final C1q binding site to *Ts*Pmy241-280aa. To further confirm whether the 40 amino acid peptide P2 can bind to C1q or not, an ELISA was performed with C1q coated on a plate, then incubated with biotinylated P2 (B-P2) and detected with streptavidin. Results showed that peptide B-P2 was capable of binding C1q in a dose-dependent manner (Fig. 4c). The same amount of B-P2 did not bind to BSA control (Fig. 4d). These findings confirm that P2 is able to bind to C1q and the 40 amino acid re sidues between ^241^Val and ^280^Ile of *Ts*Pmy (VKTQLAQQLEEARRRLEDAERERSQMQTQLHQMQLELDSI) contains the C1q- binding site.

### Inhibition of classical complement activation and complement-mediate hemolysis by peptide P2

**Fig. 5:**
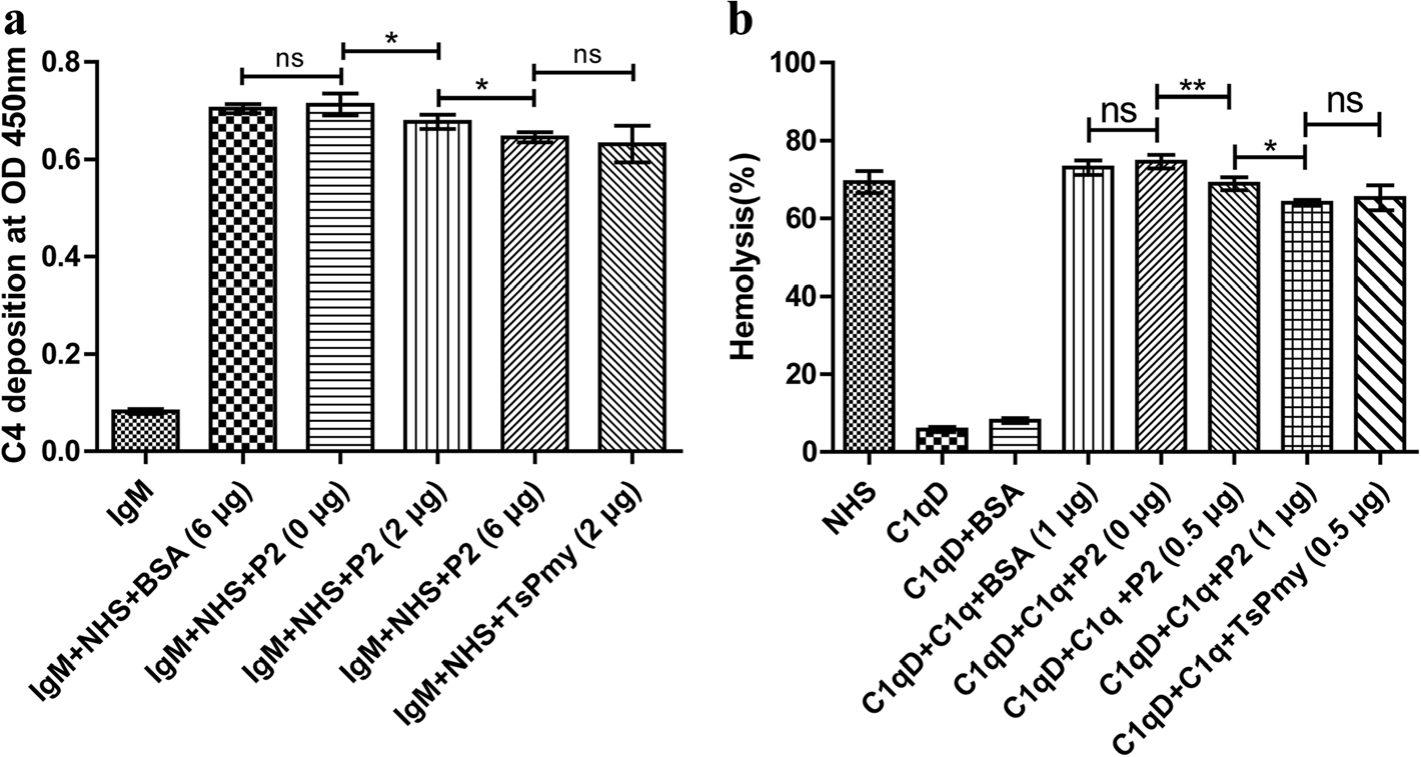
Inhibition of classical complement activation and complement-mediated hemolysis by peptide P2. **a** Inhibition of classical complement activation: normal human serum (NHS) was incubated with an increasing dose of peptide P2 (0, 2 and 6 μg), BSA (6 μg), or *Ts*Pmy (2 μg), then added to human IgM-coated plates. The C4 deposition was detected with goat anti-human C4 mAb. **b** Inhibition of complement-mediated hemolysis: C1q was pre-incubated with P2 (0, 0.5 and 1.0 μg), BSA (1 μg), or *Ts*Pmy (0.5 μg) before being added to human C1q-deficient serum (C1q D); then, sensitized fresh sheep erythrocytes (SRBC) were added. The percentage of hemolysis was compared with water induced full lysis. BSA, C1q D and NHS alone served as controls. Data are shown as mean ± SD for three independent experiments. *P < 0.05, **P < 0.01. *Abbreviation*: ns, no significant difference

To assess whether the C1q-binding peptide P2 has the ability to interfere with the human C1q complement function, IgM-C1q-initiated C4 deposition and complement-mediated hemolysis were tested. The results showed that the addition of P2 (0, 2, 6 μg) to NHS inhibited C4 deposition in a dose-dependent manner (P2 0 μg *vs* P2 2 μg, *t* = 2.566, *df* = 27, *P* = 0.016; P2 2μg *vs* P2 6 μg, *t* = 2.217, *df* = 27, *P* = 0.035). Six micrograms of P2 possessed a similar inhibitory effect to 2 μg of full-length *Ts*Pmy (P2 6 μg *vs Ts*Pmy 2 μg, *t* = 1.069, *df* = 27, *P* = 0.295). The same amount of BSA (6 μg) had no any inhibitory effect on C4 deposition (BSA 6 μg *vs* P2 0 μg, *t* = -0.496, *df* = 27, *P* = 0.624) (Fig. 5a). At a similar level to the full-length *Ts*Pmy (P2 1 μg *vs Ts*Pmy 0.5 μg, *t* = 0, *df* = 22, *P* = 1.000), the inhibitory effect of P2 on C1q-induced classical complement-mediated hemolysis was observed (P2 0 μg *vs* P2 0.5 μg, *t* = 3.801, *df* = 22, *P* = 0.001; P2 0.5 μg *vs* P2 1 μg, *t* = 2.110, *df* = 22, *P* = 0.046) (Fig. 5b). There was no significant hemolysis in the presence of C1q D serum because the classical pathway could not be activated without C1q. BSA had no inhibitory effect on complement-mediate hemolysis (BAS 1 μg *vs* P2 0 μg, *t* = -1.247, *df* = 22, *P* = 0.226) (Fig. 5b).

### Inhibition of C1q-induced chemotaxis of THP-1-derived macrophages by peptide P2

**Fig. 6:**
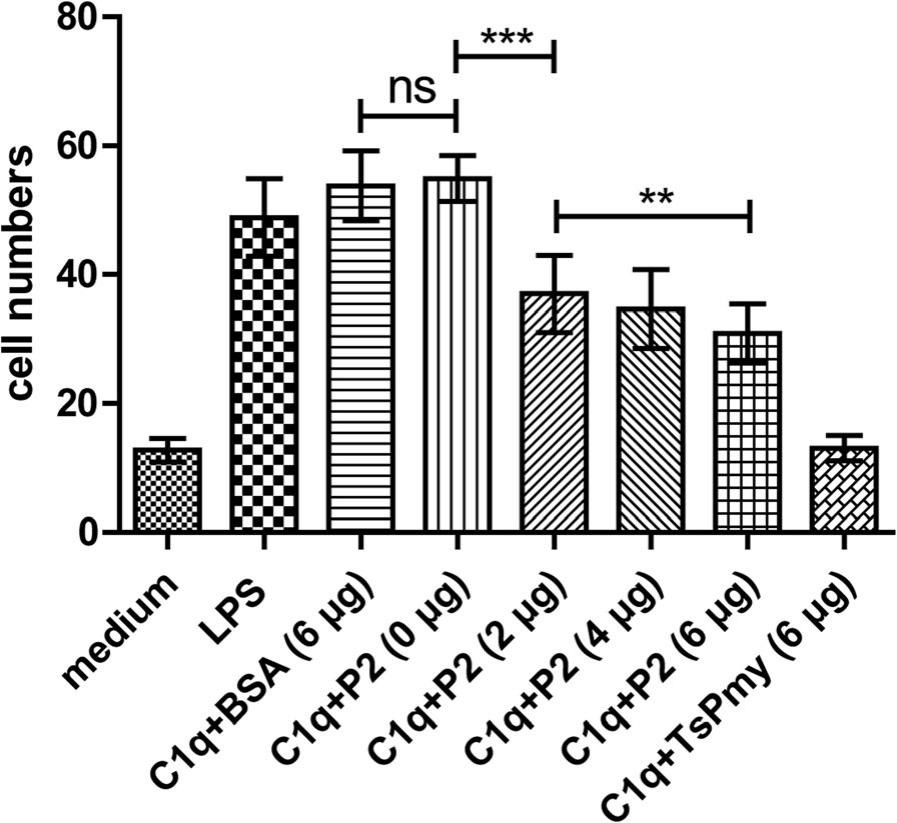
Inhibition of C1q-induced chemotaxis of THP-1-derived macrophages by peptide P2. THP-1-derived macrophages were added to the upper chamber of a 24-well-transwell. LPS (100 ng/ml), C1q (10 nM) and C1q with various doses of peptide P2 (0, 2, 4 and 6 μg), BSA (6 μg), or *Ts*Pmy (6 μg) were added into the lower chamber. The migrated cells were calculated in 10 randomly chosen fields. Data are shown as mean ± SD for three independent experiments. **P < 0.01, ***P < 0.001. *Abbreviation*: ns, no significant difference

As shown in Fig. 6, both LPS and C1q significantly attracted THP-1-derived M2 macrophages migration through the membrane (*F*_(7,103)_ = 151.081, *P* < 0.001). However, pre-incubation with peptide P2 significantly inhibited C1q-induced M2 macrophage migration through the membrane in a dose-dependent manner (P2 0 μg *vs* P2 2 μg, *t* = 9.813, *df* = 103, *P* < 0.001; P2 2 μg *vs* P2 6 μg, *t* = 9.813, *df* = 103, *P* = 0.001) (Fig. 6) as did full-length *Ts*Pmy. The same amount of BSA did not show any inhibitory effect on M2 migration (BSA 2 μg *vs* P2 0 μg, *t* = 0.629, *df* = 103, *P* = 0.531).

## Discussion

Due to the importance of the complement system in the clearance of invaded pathogens, pathogens evolve different strategies to interfere with the activation and functions of complement including the expression of some complement regulatory proteins [18]. C1q is the key target of these pathogen expressed complement regulatory proteins. In addition to *Ts*Pmy, other complement regulatory proteins include human astrovirus coat protein [10]; paramyosin from *Taenia solium* [8]; calreticulins from *Trypanosoma carassii* [11], *Brugia malayi* [13] and *T. spiralis* [3]; scabies mite inactive protease [12]; and GAPDH from *Haemonchus contortus* [14]. All of them can bind to C1q and inhibit its ability to escape C1q-initiated complement attack and to survive in the host. C1q is not only the initiation factor for the classical complement pathway, but is also involved in a number of other immunological processes through binding to immunocyte surface receptors [31–33]. A study on infective larvae of *Strongyloides stercoralis* identified that activation of the classical complement pathway promoted the adhesion of monocytes to the larval surface and reduced the motility of the larvae; notably complement C1q was a vital component [34]. However, it is still unclear how parasitic helminths evade complement attack through acting on C1q.

Paramyosin is also expressed on the surface of some helminths with key role in immunomodulation [35, 36]. The structure of paramyosin contains an alpha-helical coil with repetitive sequences of hydrophobic and charged amino acids that contributes to its strong binding affinity to many other proteins such as human collagen [9, 37, 38], calgranulin [39], IgG [37, 38], IgA [40], C1q [8], C8 [35] and C9 [35, 38], which may be related to immunomodulatory functions.

In our previous study, we identified that *Ts*Pmy of *T. spiralis* plays important roles in immunomodulation and complement evasion mainly through binding to C8/9 [5, 18] and C1q [4] as a survival strategy in the host. Binding of *Ts*Pmy to human C1q inhibited C1q-initiated classical complement activation and C1q-induced macrophages migration [4]. The present study describes the identification of the C1q binding site in *Ts*Pmy241-280aa by sequential fragment expression. Other synthesized peptides containing more amino acids (P1) or fewer amino acids (P3-P9) than *Ts*Pmy241-280aa did not show any binding activity, indicating the C1q binding domain may need both the linearized sequence and conformational structure required for binding to C1q. It is possible that P2 peptide contains fragments of ^241^Val and ^280^Ile that forms the proper conformational structure necessary for its binding activity to C1q. The subsequent classical activation pathway inhibitory assay with P2 confirmed that this peptide was able to inhibit the formation and deposition of C4, the intermediate product of C1q-induced classical activation. It also inhibited C1q-induced chemotaxis of macrophages, further confirming that P2 is not only able to bind to C1q, but also inhibit C1q induced complement activation and macrophage migration.

Even though the full-length *Ts*Pmy has been determined as a good vaccine candidate for trichinellosis [16, 19], the difficulty in expressing *Ts*Pmy as a soluble recombinant protein, in scaling-up product development, and the functional multiplicity and complexity of the whole protein, prevent *Ts*Pmy from being developed as a recombinant protein vaccine [24]. Much effort has been made to identify the protective epitope(s) of *Ts*Pmy to streamline the design process of the *Ts*Pmy epitope or multi-epitopes vaccine against trichinellosis. Two B-cell epitopes were identified on *Ts*Pmy by phage display screening with protective monoclonal antibodies against *Ts*Pmy [20, 22] and several T-cell epitopes were predicted [41]. Vaccinating with individual B-cell or T-cell epitopes induced a limited amount of protection in mice against *T. spiralis* infection [22, 41]. Strikingly, the multi-epitope subunit vaccine with a combination of B and T cell epitopes of *Ts*Pmy [24] or with an epitope from another protective antigen, *Ts*87 [22], induced much higher protection than an individual epitope or the recombinant full-length protein which correlated with higher Th1 and Th2 immune responses [22, 24]. The identification of C1q binding domain in the *Ts*Pmy N-terminus in this study, together with the C9 binding domain that has been identified within C-terminal 14 amino acid residues (^866^Val-^879^Met) [18], provides the feasible design of a multivalent peptide vaccine for preventing infection of *T. spiralis* in immunized host through efficiently interrupting worm’s ability to evade complement attack. A multi-epitope vaccine is a promising approach for vaccine design and application to prevent infections and cancer therapy [24, 42, 43], since it has been shown to increase safety and protective efficacy, with decreased side effects, immunological competition and interference of the whole vaccine molecule. *Ts*Pmy-induced protection in immunized animal should be a consequence of protective immunity induced by multiple protective epitopes. Due to the importance of *Ts*Pmy involved in the immunomodulation of host complement activation as an immune evasion strategy, inhibition of the complement binding activity of *Ts*Pmy could possibly reduce the viability of parasites in the host by vaccinating with the C1q binding site identified in this study or/and the C9 binding domain [18]. The finding of the C1q binding site in *Ts*Pmy provides another target for the design of a multivalent peptide vaccine for preventing infection of *T. spiralis* in an immunized host. The multivalent vaccine containing C1q-binding and C9-binding epitopes (domains) against *T. spiralis* infection is under investigation.

## Conclusions

Our study has identified the C1q binding domain within ^241^Val **-** ^280^Ile region of *Ts*Pmy. The synthesized peptide P2 based on this region was able to interfere with C1q-initiated complement classical activity and C1q-induced macrophage chemotaxis at a similar level to recombinant full-length *Ts*Pmy protein. This study provides molecular insight into the interaction between *Ts*Pmy and human C1q.

## Abbreviations

Ab: Antibody
B-P2: Biotinylated peptide P2
BSA: Bovine serum albumin
C1q D: C1q-deficient serum
ELISA: Enzyme-linked immunosorbent assay
His-tag: Histidine tag
mAb: Monoclonal antibody
NHS: Normal human serum
SD: Standard deviation
SEM: Standard error of the mean
*Ts*Pmy: *Trichinella spiralis* paramyosin
